# Brief Notes on the Cholera at Constantinople, 1865

**Published:** 1865-11

**Authors:** 


### Foreign Correspondence.

### BRIEF NOTES ON THE CHOLERA AT CONSTANTINOPLE, 1865.

CONSTANTINOPLE, Aug. 28th, 1865.

DEAR DOCTOR:—For nearly two months the cholera has raged with great violence in this city. My opportunities for observing the disease have been considerable. It is now four weeks since the disease appeared in that part of the city where I was laboring. Since that time, I have been in constant attendance on cholera patients, having, together with the Rev. Mr. LONG, a Methodist missionary, devoted my whole time to the care of the sick. In the notes I shall make, I will confine myself mainly to what has come under our own observation. A few remarks, by way of introduction, may help you to understand what follows.

1. The khans of Constantinople are large stone buildings, surrounding a court. These buildings vary in size, position, cleanliness, &c., &c. Some of them contain 300 rooms, and will accommodate 3000 to 4000 persons. Others contain only 30 or 40 rooms. The one in which we labored has 175 rooms, and, before the cholera broke out, had a resident population of 2000 persons. These persons are nearly all adult males, who come to Constantinople from the far interior, to do business here. A few of these club together and take a room in a khan. If in good circumstances, three or four will occupy one room; if poor, sometimes as many as fifteen or twenty persons occupy one room. No one knows how many of these khans there are in the city, but there must be several hundred. I should roughly estimate the whole number of persons resident in such khans at 200,000. It is more rather than less than this number. Many of these rooms are very damp, and the air very bad. An open sewer came into one corner of the khan where we were, and deposited its contents into a large tank or hole which had no outlet. You can imagine what exhalations arose, and still arise, from that place. From this brief description, you can easily see that a densely crowded khan is a rare field for cholera.

2. Mr. Long and I had a good room at one of these khans, where we met daily, attending the sick, administering medicine, giving advice, and making ourselves generally useful. We generally visited the patients together, consulted in regard to the treatment, in all respects sharing the labor and responsibility.

3. The natives of this country have an idea that a man cannot be considered sick until he is brought *down to his bed;* that he is well as long as he can keep on his feet. Many who have been attacked with cholera diarrhœa have kept on their feet, and sometimes even at their work, until the disease has exhausted their strength and they have come to the borders of the grave.

4. We have attended just 102 cases of those who were in bed when we called. These were bad cases, the symptoms plain; a number of them being in the collapsed state, and past hope, when we began to attend them. Of the 102, 18 have died; all the others have recovered, or are recovering. Besides these, we have given medicine to a large number, probably 350, who have come to our room with cholera symptoms, either diarrhœa or vomiting, or both. Of these, I presume, not 3 per cent have died.

5. The circumstances under which we have labored have been unfavorable, with two exceptions. As these men are away from home, and most of them poor, *proper nursing* has been impossible. I have already mentioned the dampness of the stone rooms, and the bad air; these men are not cleanly in their persons; they do not change their clothes often; lie down at night in the same clothes they have worn all day; are careless in all their habits, but especially in regard to their diet.

The two favorable exceptions are:—(1.) That the patients have generally *obeyed our directions.* Of those who have died, probably five out of six have not followed our instructions strictly. (2.) Most of our patients have been men in the *prime of life;* they have had a great deal of vital power to resist disease, and to recover after the disease is checked. We have noticed that old persons and persons affected by any other disease, especially if chronic, are prntty sure to die if attacked by cholera.

I will now give some of the symptoms, as we have observed them; then some of the remedies; and, finally, a number of illustrative cases.

*Symptoms.*

The disease appears to consist of two stages. When first attacked, the patient has a diarrhœa, this causes little or no pain; the discharges are like rice water, or clear water with little white particles in it, like the pulp of a white turnip; the discharges are few or many, according to the violence of the attack; pulse quick; mind active and restless. The diarrhœa is often accompanied by vomiting a yellowish water. When there is both diarrhœa and vomiting, the attack is more severe, and the case more dangerous, than when there is only diarrhœa. If these symptoms are not promptly checked, the strength of the patient will be exhausted in a few hours, and he will pass into the second stage.

In the second stage, there is not much diarrhœa, and but little vomiting; the patient's hands, feet, and limbs become cold; pulse feeble; skin on the fingers shrivelled; color of the hands and feet dark, as if the blood had settled in them; eyes heavy, sunken, and often turned up, showing much of the white of the eye; mind dull and insensible. In almost all cases, in both stages of the disease, there has been more or less fever, sometimes high and violent, sometimes low and heavy. The patients, almost without exception, have complained of great internal heat, and have *begged for water.* This has been true, even when their limbs have been quite cold. This raging thirst is one of the most marked symptoms of cholera, and one of the most difficult things to control. These are the most general symptoms. Of course there are exceptions, and, in some special cases, there are symptoms not mentioned at all above, generally arising from peculiarities in the individual, but in all cases the attack begins with vomiting or diarrhœa. If these are not checked, the patient will certainly die.

*Remedies.*

Our main reliance has been upon a mixture composed of equal parts of laudanum, tinct. of rhubarb, and spirits of camphor. The ordinary dose is 30 drops, but when a second dose has been necessary, we have generally doubled it. We have often given 60 drops, or a large teaspoonful, for the first dose, when the case was severe. In two cases, where there was both severe vomiting and diarrhœa, I gave 90 drops the first dose with the best effect; both patients recovered. This medicine is intended especially for the diarrhœa. When it has been difficult to check the vomiting, we have given another mixture, which we call "Mixture No. 2." It is composed of equal parts of tr. opii, tr. capsici, tr. sem. cardamoms, and tr. zingiberis. This "No. 2" has often proved very efficient: dose, 30 to 40 drops, or more, according to circumstances. We have put strong mustard plasters immediately, in almost all cases, on the feet and stomach. When the feet and arms have been cold, we have had them rubbed rapidly with rum or brandy, have applied bottles of hot water or hot bricks, and have taken other means to produce heat and a circulation of the blood in the extremities. In many cases, these external applications have had a good and decided influence. In a few cases, we have stopped the diarrhœa by injections of starch and laudanum. It has been necessary to forbid the use of water *entirely*. Many of the patients who have persisted in the use of water have died; others have been saved with great difficulty. Of course, the patient should eat nothing until the crisis of the disease has passed, and the patient begins to recover. Here has been one of our greatest troubles; the natives think that unless they take a large supply of food they cannot recover their strength. Several have thus brought on fatal relapses.

*Illustrative Cases.*

CASE I.—Hagop, an Armenian, from Yozgat. This was the first case I had. Age, 25. Was in the second stage when I first saw him; had been down 24 hours with diarrhœa and vomiting; had taken no medicine; had severe cramps in his legs; hands and feet cold and dark-colored. I gave him large doses of mixture No. 1 and brandy; put two men to rubbing his legs and arms; pulse very low; we checked the vomiting, and the rubbing removed the cramp. I worked over him three hours; left him, towards evening, to go home. Soon after I had gone, his friends called a barber and bled the patient, and he died in a short time. (In this country, barbers bleed and draw teeth.) I felt special interest in this case, as he was an only son. The old father was with him, and begged hard that we should try to save his son's life. The immediate cause of his death was the bleeding.

CASE II.—Marderos, a boy, 14 years old, Armenian; was passing into second stage, or collapse, when I first saw him; had a high fever, great heat internally, but feet and hands soon became cold; begged incessantly for water. Gave mixture No. 1, checked the diarrhœa, put mustard plasters on feet and stomach, rubbed him a great deal with rum; two men worked over him constantly for several hours. Next morning he was better, but had a low fever. He recovered slowly, but is now well.

CASE III.—Sdepan, brother of Marderos. He assisted in the care of M., and became very much exhausted; was taken with violent vomiting and purging, which had continued three hours when I saw him. Gave him a strong dose of No. 1, which he immediately threw up; in a few moments, gave him a second dose, followed by a little brandy; made him lie flat on his back; he retained this dose, which checked the disease. He recovered with very little fever; in three days he was about his room.

CASE IV.—A *hamal*—burden-bearer. (As I shall mention this class of men often, let me say that they are men of immense physical strength; the loads they sometimes carry on their backs are enormous, weighing from 200 to 600 lbs. Of course they are men of good health. Most of them are Armenians, from Van.) A man of great size and strength, in second stage; hands and feet cold and blue; voice thick; mind dull; his whole body stiff and hard; had taken no medicine; was lying between an open window and an open door, with a strong draft of wind passing over him; his companions were rubbing his feet and hands. I considered him beyond hope. Gave him 60 drops of mixture No. 1, and a large dose of brandy; left brandy to be given him every half-hour during the night. Next morning, went to his room, expecting to find him dead, when he saluted me with a "good morning, Sir!" His companions said that, about midnight, he "opened his eyes, sat up, and asked for a bowl of water to wash his face." He recovered without any relapse, and with very little fever.

Case V.—Another *hamal;* passing into collapse when I saw him; gave the usual remedies, and put mustard plasters on his feet, arms, and stomach; disease checked, but his thirst was so great that in the night he crawled to a jug of water and drank freely, which brought on a relapse; both the diarrhœa and vomiting returned. The next day he was very low, stupid, dull fever; pulse very feeble. Gave him brandy in large doses; put men to rubbing his limbs with rum. Symptoms checked, but his strength was gone. The third morning he was alive, but his body was covered with little pimples and festers; very feverish; persisted in drinking water during the previous night; had had two discharges. Died on the third night. I feel quite confident that he would have recovered if he had obeyed our directions.

Case VI.—An Armenian carpenter, old man; while at work on a building was taken with a diarrhœa, but slight; seemed struck down by heat; brought into the khan about 3 P.M. I gave him mixture No. 1. He had a burning fever; great thirst; begged for water; respiration very difficult; had no more diarrhœa, but died the same night, about nine hours after he was first attacked.

Case VII.—A Bulgarian boy, 17 years old; had been sick when we first saw him, about ten hours; had violent vomiting and purging; was entirely exhausted; eyes turned back and sunken; feet cold; skin shrivelled; his whole appearance indicating that death would follow very soon. Gave him a dose of mixture No. 2, which he immediately threw up, with a great quantity of yellowish water. Gave him another dose, with two teaspoonfuls of brandy; put mustard plasters on feet, arms, and stomach; disease was checked, but typhus followed. After a sickness of thirteen days he was able to leave his room.

Case VIII.—A Greek tobacconist, 25 years of age; strong, very healthy; attack sudden and violent; had diarrhœa and vomiting. We did not have the exclusive charge of this case; when others had attended him for some time, and there seemed no hope of his recovery, we were called. We applied mustard plasters to his feet, arms, and stomach, gave him mixture No. 1 and some brandy; his feet were cold; applied hot bricks, bottles of hot water, &c., to his limbs, and had them rubbed. He was attacked early in the morning; when we left him, about 5 P.M., there seemed some hope of his recovery, as the discharges had ceased. As we left the room, we met a Jew doctor, who said he must be *bled*. We told him that if he was bled, he would die. After we left, the Jew M.D. bled him, and he died almost immediately.

Case IX.—A Russian Armenian, an old man, had nc one to take care of him; when I found him he had a jug of water by his side, from which he drank freely; he had both vomiting and diarrhœa; both were checked by the usual methods, but he would not desist from drinking water. The next morning he was alive, but the jug of water, which I had removed, was again by his side. His voice was thick and all his symptoms were bad. He was entirely alone, and so he died, before dark, having been sick nearly two days.

Case X.—Turkish *khoja*, or teacher, 35 years old, strong, in the prime of life; passing into the second stage when called. Mixture No. 1, mustard plasters, brandy, and rubbing saved him. The muscles of his legs were cramped, as if in knots.

A very marked illustration of the influence of medicine—we only gave him medicine once.

CASE XI.—Turk, young man; had been sick twelve hours when we saw him; did not know how many discharges he had had, he had not counted them, thought 15 or 20; pulse low; whole system much depressed and exhausted. The father, in his ignorance, had given him as much water as he wanted, but said, "as soon as he drinks he either vomits or his bowels move." 60 drops of mixture No. 1; mustard plaster; (the old man made a plaster long enough to go clear around the boy's body.) When we returned, after seeing him the first time, and found such a monster plaster ready, we laughed not a little, but told him to clap it on, which he did. The boy recovered.

CASE XII.—An Arab, 25 years old; symptoms very bad; diarrhœa, vomiting; feet becoming cold. We gave him a large dose of No. 1, and a good dose of brandy; ordered the mustard plasters and rubbing. In three days he was about his business.

CASE XIII.—A poor Armenian boy, about 17 years of age; passing into second stage. Dr. PRATT, an experienced missionary physician, saw him; said he thought there was little use in giving him medicine. Ordered a strong dose of No. 2, with brandy in large doses, every twenty minutes, until he began to improve. Put on the mustard. Much to our surprise, he recovered.

I might go on and fill several sheets with such particulars, but have not the time, and perhaps it would add nothing to the value of these notes. I fully intended to give you the particulars of at least 25 cases. All I can now do, is to "close with a few remarks."

1. Pardon the imperfection of what I have written. I know but little about medicine and medical terms. Perhaps there will be nothing new or peculiar in all I have said. I have written only as an *observer* of actual cases of cholera, and not at all as one competent to treat the disease, or to describe it.

2. My observations have convinced me that the operations of the mind have great influence in bringing on, or in keeping off the disease. I have known several instances where fear seems to have been the principal or exciting cause of the disease, while very few are attacked whose minds are entirely free from fear and anxiety.

3. When the remedies are applied *early* in the first stage of the disease, it can be easily controlled, as a general rule. When not till the patient is in the second or malignant stage, success is very doubtful.

4. From the time the cholera first came here, there has been an impression that rum, brandy, &c., would keep it off; the consequence has been, that about three-fourths of the population went furiously to drinking bad New England rum, and all other kinds of liquor they could lay their hands upon. The effect has undoubtedly been very bad.

5. It is impossible to estimate the number of deaths by cholera, in this city, during the past two months. The published official statements are notoriously false, being far below the truth. One of our friends, a medical man, who is more or less connected with the Government, says he was one day in the office of the Grand Vizier, or Secretary of State, and saw there the true official summary of the whole number of deaths for the previous Monday. This number included all the suburbs of the city, and amounted to 1879, for that day alone This was some days after the crisis of the pestilence had passed. Many other facts have come to our knowledge to confirm his statement, but I will not mention them now. My impression is, that if we should take 500 as the average number of deaths per day, for 60 days, we should be below, rather than above, the truth. This gives 30,000 as the whole number. Many place it as high as 58,000. The desire of the Government to prevent a panic, has kept them from publishing the true returns. It may be doubted whether the Government itself knows the whole truth.

